# Skeletal Muscle Spheroids as Building Blocks for Engineered Muscle Tissue

**DOI:** 10.1021/acsbiomaterials.3c01078

**Published:** 2023-12-19

**Authors:** Nicholas Johnson, Andrea C. Filler, Akash Sethi, Lucas R. Smith, J. Kent Leach

**Affiliations:** † Department of Orthopaedic Surgery, UC Davis Health, Sacramento, California 95817, United States; ‡ Department of Biomedical Engineering, UC Davis, Davis, California 95616, United States; § Department of Molecular and Cellular Biology, UC Davis, Davis, California 95616, United States; ∥ Department of Neurobiology, Physiology and Behavior, UC Davis, Davis, California 95616, United States

**Keywords:** spheroids, skeletal muscle, bioprinting, muscle engineering, hydrogel

## Abstract

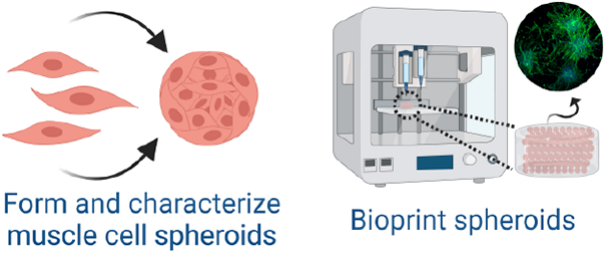

Spheroids exhibit enhanced cell–cell interactions that facilitate improved survival and mimic the physiological cellular environment *in vivo*. Cell spheroids have been successfully used as building blocks for engineered tissues, yet the viability of this approach with skeletal muscle spheroids is poorly understood, particularly when incorporated into three-dimensional (3D) constructs. Bioprinting is a promising strategy to recapitulate the hierarchical organization of native tissue that is fundamental to its function. However, the influence of bioprinting on skeletal muscle cell spheroids and their function are yet to be interrogated. Using C2C12 mouse myoblasts and primary bovine muscle stem cells (MuSCs), we characterized spheroid formation as a function of duration and cell seeding density. We then investigated the potential of skeletal muscle spheroids entrapped in alginate bioink as tissue building blocks for bioprinting myogenic tissue. Both C2C12 and primary bovine MuSCs formed spheroids of similar sizes and remained viable after bioprinting. Spheroids of both cell types fused into larger tissue clusters over time within alginate and exhibited tissue formation comparable to monodisperse cells. Compared to monodisperse cells in alginate gels, C2C12 spheroids exhibited greater MyHC expression after 2 weeks, while cells within bovine MuSC spheroids displayed increased cell spreading. Both monodisperse and MuSC spheroids exhibited increased expression of genes denoting mid- and late-stage myogenic differentiation. Together, these data suggest that skeletal muscle spheroids have the potential for generating myogenic tissue via 3D bioprinting and reveal areas of research that could enhance myogenesis and myogenic differentiation in future studies.

## Introduction

Skeletal muscle tissue engineering has the potential to address key clinical and societal challenges, such as traumatic muscle injury, our fundamental understanding of skeletal muscle development and disease, and the future of meat production via cultivated meat.^1−3^ Preclinical animal models and monolayer cultures have driven our understanding of skeletal muscle development and function. However, two-dimensional (2D) cell cultures lack complex cell–cell and cell–extracellular matrix (ECM) interactions that provide essential biochemical and biomechanical signals directing cell function *in vivo*.^4^ Engineered tissue should model the complexity of native muscle, which has led to the innovation of advanced fabrication methods such as casting ECM-derived hydrogels around posts to introduce uniaxial tension and more scalable techniques like electrospinning and 3D bioprinting.^5^

Spheroids are dense cellular aggregates that are promising building blocks for tissue engineering due to their increased cell–cell interactions, upregulated cytokine production, retention of endogenous ECM, as well as enhanced cell survival *in vitro* and *in vivo* compared to monodisperse cells.^4,6−9^ Spheroids have been fabricated from a variety of cell types,^7,10,11^ yet literature on skeletal muscle spheroids is sparse.^12^ Aggregation of muscle cells into spheroids has largely focused on understanding the behavior of primary muscle cells,^13−17^ but the application of this approach in tissue engineering lags behind other tissue types such as bone,^18,19^ cardiac,^20,21^ and adipose,^22,23^ among others. Studies using immortalized cell lines in muscle spheroids have shown promising results with regard to cell survival and differentiation. For example, C2C12 spheroids, once dissociated, exhibited higher proliferation, upregulated MyoD expression, and enhanced myogenic potential in both 2D and 3D culture.^16^ Furthermore, C2C12 spheroids possess upregulated myogenic markers compared to monodisperse cells and can differentiate into aligned myotubes on electrospun substrates.^17^ However, little is known about how muscle cell spheroids function in contiguous matrices, such as bioinks, representing a key information gap in the field.

Bioprinting is a promising biofabrication technique for generating structured tissue due to the potential to precisely pattern multicellular constructs of relevant cell types (i.e., myoblasts, fibroblasts, adipocytes, and their progenitor cells).^1,24^ Most bioprinting applications use monodisperse cells,^25−27^ which require disruption of essential cell–cell and cell–matrix interactions when cells are enzymatically removed from the culture substrate. Little is known about the interplay of shear forces during bioprinting and cell viability of spheroids, although much has been reported for monodisperse cells.^28,29^ Additionally, a growing body of research confirms the benefits of retaining endogenous ECM with associated cells to recapitulate the native extracellular environment and support cell function.^30,31^ Synthetic biomaterial scaffolds are highly tunable, yet often lack the complex biophysical and biochemical cues provided by native ECM, hence, exposing a key limitation of bioprinting that spheroids may address.

We hypothesized that skeletal muscle cell spheroids will function as building blocks of muscle tissue when embedded in 3D microenvironments. In this study, we bioprinted skeletal muscle spheroids to assess cell function and tissue-forming potential compared to monodisperse cells. We utilized 3D bioprinting as a proof of concept to determine whether bioprinting may adversely affect cell viability. Experiments were first performed using C2C12 spheroids before translation to more clinically and culinarily relevant primary bovine MuSCs. These data demonstrate that skeletal muscle spheroids are promising building blocks for muscle tissue and validate 3D bioprinting as a compatible fabrication technique.

## 2 Materials and Methods

### 2.1 C2C12 Cell Culture

C2C12 mouse myoblasts (ATCC CRL-1772, Manassas, VA) were expanded under standard cell culture conditions (37 °C, 21% O_2_, 5% CO_2_) in Dulbecco’s Modified Eagle Medium (DMEM) (ThermoFisher, Waltham, MA) supplemented with 10% fetal bovine serum (FBS) (Bio-Techne, Minneapolis, MN) and 1% penicillin (10 000 U/mL) and streptomycin (P/S) (10 mg/mL) (Gemini Bio Products, West Sacramento, CA) until use at passages 3–7. C2C12s were seeded at 5000 cells/cm^2^, media was refreshed every 2–3 days, and cells were passaged before reaching 80% confluency using Trypsin EDTA (0.25%) (ThermoFisher). For differentiation, cells were cultured in DMEM supplemented with 2% horse serum (ThermoFisher) and 1% P/S under standard cell culture conditions.

### 2.2 Primary Bovine Bell Isolation and Culture

Primary bovine MuSCs were isolated from freshly slaughtered Angus cow semitendinosus muscle received from the UC Davis Meat Lab by adapting a previously reported protocol.^32^ Briefly, bovine tissue was submerged in 70% ethanol, and then ∼1 g of muscle was minced. The minced tissue was transferred into a collagenase solution (2000 units/mL CLSAFA, Worthington Biochemical, Lakewood, NJ) and incubated at 37 °C under continuous rotation on a MACSmix Tube Rotator (Miltenyi Biotec, Bergisch Gladbach, Germany) for 1 h with further mechanical dissociation at 30 min intervals using the gentleMACS dissociator (Miltenyi Biotec). Debris was removed by filtration through a 100 μm nylon cell strainer, red blood cell lysis using an ammonium–chloride–potassium (ACK) lysis buffer, and further filtered through a 40 μm cell strainer. Suspended cells were enriched for satellite cells using magnetic-activated cell sorting (MACS) and the satellite cell isolation kit (Miltenyi Biotec) following manufacturer’s protocols. Satellite cells were validated by immunofluorescence for Pax7 (Developmental Studies Hybridoma Bank, Iowa City, IA).

Cells were expanded under standard cell culture conditions in Ham’s F10 media supplemented by 20% FBS, 5 ng/mL basic Fibroblast Growth Factor (bFGF) (R&D Systems, Minneapolis, MN), 1% P/S, and Amphotericin B (25 μg/mL) (PSA) (Sigma-Aldrich, St. Louis, MO). Differentiation media was composed of DMEM (1 g/L glucose) supplemented with 2% FBS and 1% PSA. Cells were seeded at 2000 cells/cm^2^ for proliferation on culture flasks coated with purified bovine type I telocollagen (TeloCol-3, Advanced Biomatrix, Carlsbad, CA), media was refreshed every other day, and cells were used between passages 1–4 for maintenance of differentiation capacity.

### 2.3 Spheroid Formation

Spheroids were formed using a forced aggregation method as we reported.^33^ Spheroids were produced by seeding each well with 500, 1000, 2000, 5000, or 10 000 cells. Plates were then maintained in static culture conditions for 48 h to enable spheroid compaction. Spheroid size was determined by measuring the diameter of at least 12–16 individual spheroids, formed in four different wells per experimental replicate, in ImageJ.

### 2.4 Bioink Preparation

Ultrapure MVG sodium alginate (viscosity > 200 mPa·s, MW > 200 kDa, and G/M ratio ≥ 1.5; Pronova Novamatrix, Norway) was oxidized to 1% to facilitate degradation by hydrolysis^34,35^ and covalently modified with arginine–glycine–aspartic acid peptide (GGGGRGDSP; Peptide 2.0, Chantilly, VA), as we reported^36^ such that each alginate chain had a degree of substitution (DS) of 2. The alginate was then dialyzed (3.5 kDa MWCO, Spectrum Chemical, New Brunswick, NJ) in ultrapure water for 4 days, sterile filtered, lyophilized for 4–7 days, and subsequently resuspended in sterile PBS to 3.5 w/v%. To prepare the bioink, the 3.5% alginate was pre-crosslinked for 2 min at a 7:3 ratio with 50 mM CaCl_2_ (MilliporeSigma, Burlington, MA) using the Luer lock mixing method.^37^ After pre-crosslinking, the final concentration of the alginate bioink was 2.45 w/v%.

### 2.5 Cell and Spheroid Bioprinting

CaCl_2_ solutions of 50 and 100 mM were prepared in differentiation media and sterile filtered. Monodisperse cells and spheroids were collected, centrifuged, and resuspended at 10, 20, or 50 × 10^6^ cells/mL in 3.5% alginate warmed to 37 °C. The cell–alginate mixture and 50 mM CaCl_2_ solution were mixed as described above to generate cell-laden bioinks. We used an Allevi 2 bioprinter (3D Systems, Philadelphia, PA) to print 4 × 1.5 mm cylinders at 6 mm/s for each condition, which required air pressure between 15 and 25 psi. Prints were conducted with Allevi plastic tips with a 23 gauge and 6.35 mm long stainless-steel needle (Allevi). To crosslink the alginate disks after bioprinting, samples were submerged in 100 mM CaCl_2_ for 10 min, then flipped and submerged for another 5 min. A basic schematic of the process to formulate the alginate bioink and make bioprinted and cast gels is shown in Figure S1.

### 2.6 Cast Spheroid Gels

Spheroids embedded within alginate via casting in silicone molds were used as a positive control to characterize spheroid viability postprinting. Silicone molds were made by cutting 4 mm diameter disks out of 1.5 mm thick silicone mats by using a 4 mm biopsy punch and then autoclaved. Dialysis membrane was sterilized in 70% ethanol for 30 min, rinsed twice in PBS and placed on a glass plate, followed by the silicone mold. To fabricate the alginate molds, the spheroid-laden bioink was prepared as described above and injected into each 4 mm cutout. The molds were covered with a second dialysis membrane, and immersed in 100 mM CaCl_2_ for 5 min, flipped, cross-linked for another 5 min, then removed from the molds and submerged for another 5 min.

### 2.7 Live/Dead Assay

Cell viability was analyzed by live/dead assay per the manufacturer’s protocol (ThermoFisher). Spheroids were collected from the agarose inverted molds, stained, and plated on a glass dish for imaging. When bioprinted constructs were characterized, the entire gel containing monodisperse cells or spheroids was stained. Live/dead images were taken using 10× and 20× objectives on a Leica confocal microscope (Leica STELLARIS, Leica Camera AG, Wetzlar, Germany). Quantification of viability was performed by measuring the relative area of live stain compared to the total stained area using ImageJ.

### 2.8 Metabolic Activity, Cell Proliferation, and Apoptotic Activity

Metabolic activity was determined via the alamarBlue assay. Spheroids or spheroid-laden constructs were immersed in alamarBlue reagent (ThermoFisher) diluted 1:10 in media for 3 h. Media fluorescence was measured using a Synergy HTX Multi-Mode Plate Reader (Biotek, Winooski, VT). Samples were collected in passive lysis buffer (Promega, Madison, WI) and stored at −20 °C. Samples were then sonicated, and total double-stranded DNA (dsDNA) was measured using a Quant-iT PicoGreen dsDNA Assay Kit (Invitrogen, Waltham, MA). Apoptosis was quantitatively measured using a Caspase-Glo 3/7 assay (Promega). The total metabolic activity and apoptotic activity were then normalized to DNA content for each sample.

### 2.9 Histology

Cell morphology and myogenic differentiation were characterized by confocal fluorescence microscopy. Alginate gels were fixed at 4 °C overnight in 4% paraformaldehyde (PFA), rinsed two times with PBS, permeabilized with 0.5% Triton X-100 (Sigma-Aldrich), and stained with Alexa Fluor Phalloidin 488 (1:40, ThermoFisher) for 1 h followed by DAPI (1:400, ThermoFisher) for 10 min. Images of stained samples were taken on a confocal microscope (Leica STELLARIS) using 10×, 20×, and 40× (water immersion) objectives.

Myogenic differentiation of cells in printed constructs was interrogated by immunofluorescence. Cell-laden alginate gels were fixed overnight in 4% PFA at 4 °C. After rinsing with PBS, gels were sequentially dehydrated in 30%, 50%, and 70% (v/v) ethanol for 30 min in each solution. Samples were paraffin-embedded and sectioned at 8 μm. For immunostaining, slides were rehydrated and exposed to heat-mediated antigen retrieval with sodium citrate buffer (pH 6). Samples were permeabilized with 0.1% Triton X-100 for 10 min at room temperature then incubated in blocking buffer consisting of 10% goat serum (Cell Signaling Technology, Danvers, MA) and 10 mg/mL Bovine Serum Albumin (BSA) (Sigma-Aldrich) for 30 min at room temperature. Samples were then incubated with antifast myosin skeletal heavy chain primary antibody (ab91506, 1:100, Abcam, Cambridge, UK) at 4 °C overnight. Next, the sections were incubated with goat antirabbit Alexa Fluor 594 IgG (H&L) secondary antibody (ab150080, 1:250, Abcam) at 4 °C for 4 h. Cell nuclei were counterstained with DAPI for 30 min at room temperature. Stained sections were mounted with glass coverslips using VectaMount mounting medium (Vector Laboratories, Newark, CA). Fluorescent images were taken using a confocal microscope (Leica STELLARIS) using 20× and 40× (water immersion) objectives. Quantification was performed by measuring the area of each stain relative to DAPI area using ImageJ.

### 2.10 qPCR

Bioprinted samples with monodisperse cells and spheroids were collected, immersed in 400 μL of TRIzol Reagent (Invitrogen), and homogenized by using a sonicator (Sonics, Newton, CT). RNA was isolated following TRIzol reagent instructions as per the manufacturer. 800 ng of RNA was reverse transcribed using the QuantiTect Reverse Transcription Kit (Qiagen, Hilden, Germany) and diluted to a final concentration of 12 ng/μL. qPCR was performed using Taq PCR Master Mix (Qiagen) in a QuantStudio 6 Pro real-time PCR system (ThermoFisher). Mouse specific primers for *Gapdh* (Mm99999915_g1), *Myod1* (Mm00440387_m1), and *Myog* (Mm00446194_m1) and bovine specific primers for *RPS15A* (Bt03229083_g1), MYOD1 (Bt03244740_m1), MYOG (Bt03258929_m1), and MYHC3 (Bt03258391_m1) were purchased from ThermoFisher. Critical threshold values (Ct) were quantified for each gene of interest, and the ΔCt for each sample was quantified by subtracting the sample’s Ct value of the housekeeping gene, Gapdh or RPS15A, for mouse and bovine samples, respectively. The ΔΔCt value of each sample was quantified by subtracting the average ΔCt value of the day 1 monodisperse group. Expression values for each gene were then presented as 2^–ΔΔCt^.

### 2.11 Statistical Analysis

Data are presented as means ± standard deviation. All experimental results represent at least three independent experiments unless noted. Data points are reported as the mean of technical replicates measured in triplicate for each independent experiment, unless otherwise noted. The Prism 9 software (GraphPad, San Diego, CA) was used to perform two-way ANOVA followed by Tukey’s multiple comparison test, with *p* < 0.05 considered as significant. Outliers within sample sets were characterized via Grubbs test where α = 0.05. Groups with different letters denote significance (*p* < 0.05), while groups that share a common letter are not statistically significant.

## 3 Results

### 3.1 C2C12 Spheroids Exhibit Similar Compaction Rate and Viability

**Figure 1:**
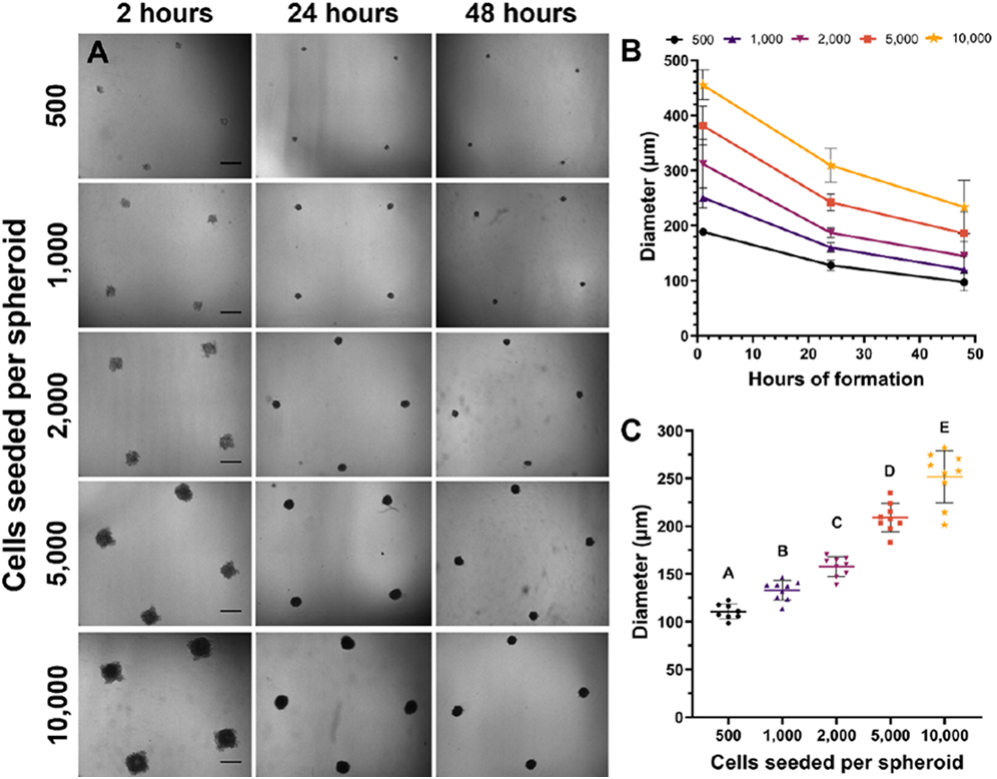
C2C12 spheroids compact over 48 h. (A) Brightfield microscopy of spheroids seeded with 500, 1000, 2000, 5000, and 10 000 cells in agarose microwells (scale bar represents 500 μm). (B) Quantification of spheroid diameter during compaction (*n* = 4). (C) Spheroid diameter for increasing spheroid cell counts measured at 48 h (*n* = 9). Groups with different letters denote significance (*p*< 0.05), while groups that share a common letter are not statistically significant.

We first characterized the size and viability of C2C12 myoblast spheroids with increasing cellular content. Spheroids seeded with 500–10 000 cells were formed via forced aggregation in nonadherent agarose microwells and imaged over 48 h (Figure 1A). Cells began forming into loose, but distinct spheroids within 2 h after seeding (Figure 1A, left), and all spheroids compacted by about 45% over 48 h (Figure 1B). The majority of compaction occurred within the first 24 h. After 48 h, C2C12 spheroid diameters were 115 ± 15.5, 132.6 ± 10.2, 157.5 ± 10.3, 208.7 ± 15, and 251.6 ±27.3 μm for spheroids seeded with 500, 1000, 2000, 5000, and 10 000 cells, respectively (Figure 1C).

**Figure 2:**
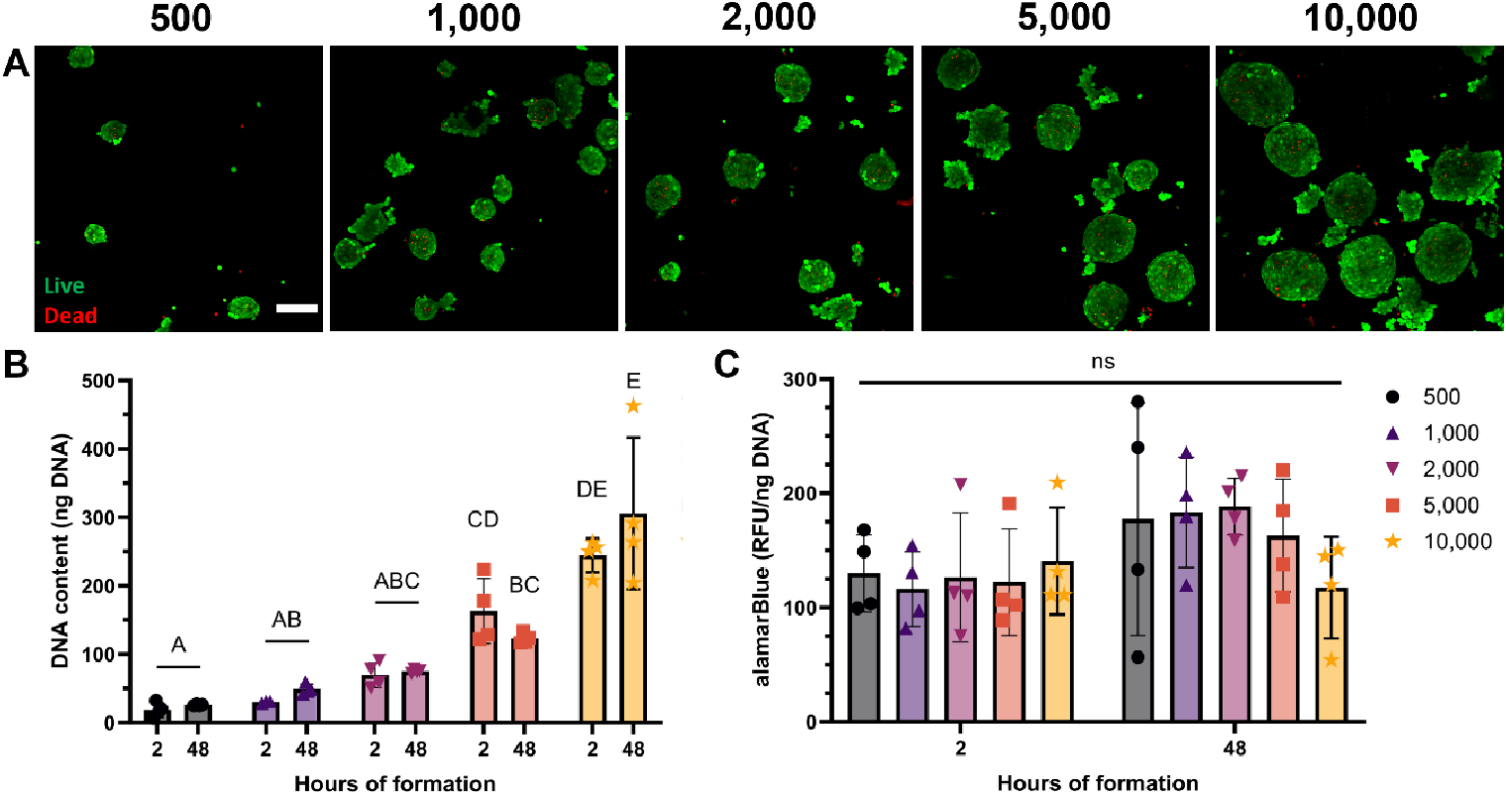
Metabolic activity remains constant among the studied spheroid diameters. (A) Confocal z-stack max projections of live/dead assay for spheroids seeded with 500, 1000, 2000, 5000, and 10 000 cells (scale bar represents 150 μm). Calcein AM (green) corresponds to live cells, and propidium iodide (red) corresponds to dead cells. (B) Total DNA content (*n* = 4). (C) Metabolic activity (*n* = 4). Groups with different letters denote significance (*p*< 0.05), while groups that share a common letter are not statistically significant.

Spheroids did not develop a necrotic core, and we did not observe substantial differences in cell viability between groups 2 days after spheroid formation (Figures 2A and S2A). Some dead cells were present within the spheroids, but as spheroid diameter increases, so does the background fluorescence for out-of-plane cells, making image quantification less reliable. DNA content increased with the increase in cell number, as expected, but did not scale proportionally with cell number (Figure 2B). The agarose molds used in this study do not perfectly fill the well plate, which allows some cells to escape the micropatterned microwells and attach to the nontreated plastic surface underneath the agarose molds rather than being incorporated into spheroids.^38^ As more cells were added to the wells to create larger spheroids, more cells likely escaped below the agarose in those wells, which may account for this discrepancy.

Regardless of the spheroid diameter, metabolic activity was relatively consistent at both time points (Figure 2C). Furthermore, we did not observe differences in Caspase 3/7 activity, an indicator of apoptosis, among all conditions (Figure S2B). Overall, these data confirm that there is no substantial difference in cell viability between spheroids with diameters less than 250 μm. Therefore, we used spheroids seeded with 5000 cells for the remaining experiments, as they are easier to handle than smaller spheroids and can fit through most needles used in bioprinting including our 23-gauge needle (inner diameter = 330 μm).

### 3.2 Spheroids Remain Viable After 3D Bioprinting

**Figure 3:**
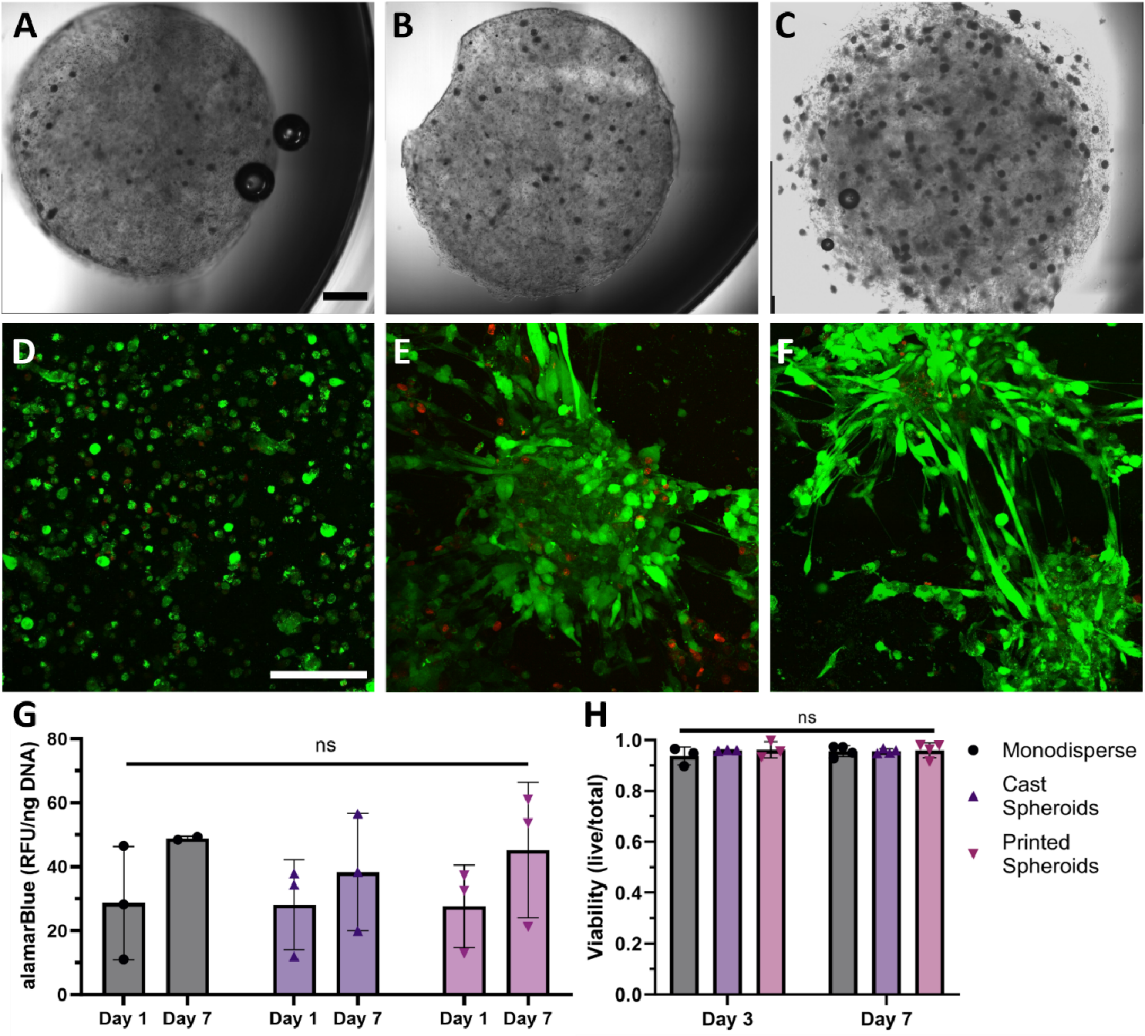
Spheroids remain intact and viable after 3D bioprinting. (A–C) Bioprinted alginate disks containing spheroids at 10, 20, and 50 × 10^6^ cells/mL, (scale bar represents 1 mm). (D–F) Live/dead confocal images at day 3 of bioprinted monodisperse cells, cast spheroids, and bioprinted spheroids at 50 × 10^6^ cells/mL (scale bar represents 150 μm). (G) Metabolic activity of spheroids in different conditions (*n* = 3). (H) Quantification of cell viability within alginate constructs after 3 and 7 days calculated by dividing area of live stain/total (*n* = 3, where *n* represents images from independent samples).

We tested the influence of spheroid density in the bioink to alter interspheroid spacing, as spacing between spheroids can influence both cell migration and paracrine signaling.^39,40^ We encapsulated C2C12 spheroids in oxidized, RGD-modified alginate and precross-linked with 50 mM CaCl_2_ in a 7:3 ratio to create a viscous bioink with the rheological and shear thinning characteristics needed for bioprinting, as demonstrated by previous data.^37^ Cylindrical constructs were printed with increasing cell densities of 10, 20, and 50 × 10^6^ cells/mL, respectively (Figure 3A–C). Spheroids remained intact and were dispersed throughout the construct, although the distribution of spheroids was heterogeneous. We selected 50 × 10^6^ cells/mL for further experiments to increase interspheroid interactions, as lower seeding densities would increase the heterogeneity within alginate gels.

To assess spheroid viability postbioprinting, we printed cylindrical constructs with monodisperse cells at the same cell density to serve as a control (Figure 3D). Additionally, alginate constructs with embedded spheroids were fabricated via casting in a mold (Figure 3E) and compared to bioprinted spheroids (Figure 3F) to determine whether bioprinting adversely affects the spheroid viability. We did not observe any appreciable differences after 3 or 7 days (Figures 3H and S2C). Metabolic activity for bioprinted spheroids was similar to monodisperse and cast spheroid samples at each time point, and data show an upward trend in alamarBlue staining for each group at 7 days (Figure 3G). Collectively, these data indicate that the mechanical forces endured by spheroids seeded with 5000 cells during bioprinting do not negatively influence cell viability.

### 3.3 C2C12 Spheroids Exhibit Enhanced Myogenic Differentiation within Alginate Bioink

**Figure 4:**
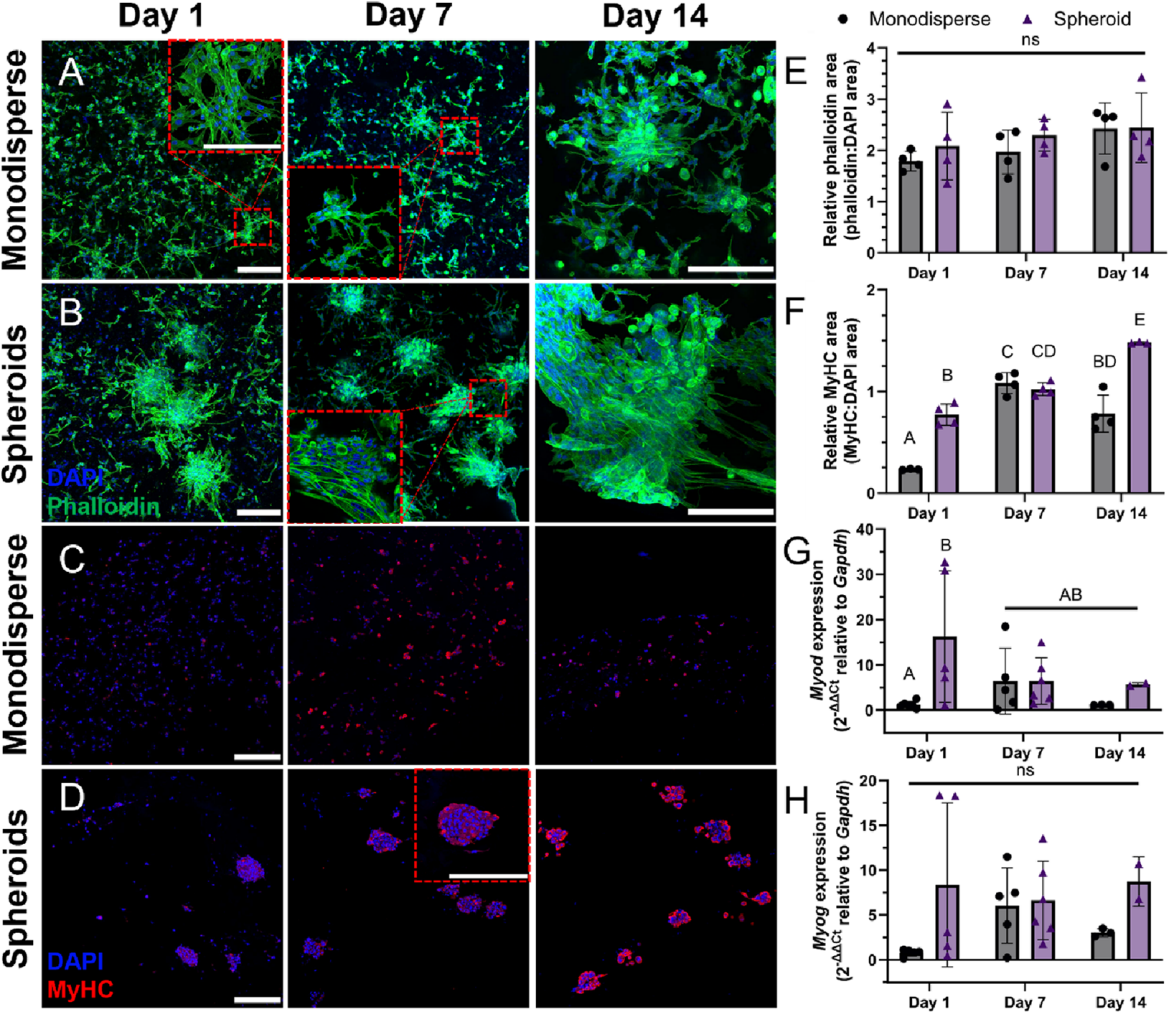
C2C12 spheroids exhibit increased cell spreading and differentiation within alginate hydrogels. Confocal z-stack projection micrographs of (A) monodisperse cells and (B) spheroids bioprinted in alginate bioink and differentiated over 14 days (scale bars represent 200 μm). DAPI (blue) stain for the nucleus and Phalloidin (green) for F-actin. Immunostaining of bioprinted (C) monodispersed cells and (D) spheroids with DAPI (blue) and myosin heavy chain (red) over 14 days (scale bars represent 200 μm). Quantification of the ratio of (E) Phalloidin:DAPI and (F) MyHC:DAPI stain area and gene expression of (G) *Myod* and (H) *Myog* over 14 days (*n* = 2–6, where *n* represents an independent sample). Groups with different letters denote significance (*p*< 0.05), while groups that share a common letter are not statistically significant.

To determine how cells interact and differentiate within a 3D microenvironment, cylindrical constructs were printed with either monodisperse C2C12s or spheroids at 50 × 10^6^ cells/mL. Constructs were cultured for up to 14 days in differentiation media and analyzed via confocal microscopy, immunohistochemistry, and qPCR. Both monodisperse cells and spheroids exhibited substantial spreading after 24 h of incubation (Figure 4A,B), but showed no significant changes over time (Figure 4E). Monodisperse cells tended to migrate toward the edges of the constructs, where we observed the greatest spreading and fusion for both samples. Cells on the spheroid periphery migrated into the surrounding matrix and tended to bridge the gap between spheroids in close proximity. Myosin heavy chain (MyHC) staining revealed that spheroids expressed more MyHC over time, whereas MyHC staining in monodisperse samples did not exhibit the same temporal progression (Figure 4C,D,F). To further characterize differences in differentiation between monodisperse cells and spheroids, we measured the gene expression of *Myod* and *Myog* (Figure 4G,H). Spheroids exhibited reduced *Myod* and relatively stable *Myog* expression over 14 days, which, when paired with increasing MyHC protein expression, indicates progression toward myogenic differentiation. Monodisperse cells exhibited similar levels of gene expression, indicating maturation, yet the trend over time was less consistent. In general, spheroids exhibited increased markers of myogenic differentiation on days 1 and 14 within gels compared to monodispersed cells.

### 3.4 Primary Bovine MuSC Spheroids Form Analogously to C2C12s

**Figure 5:**
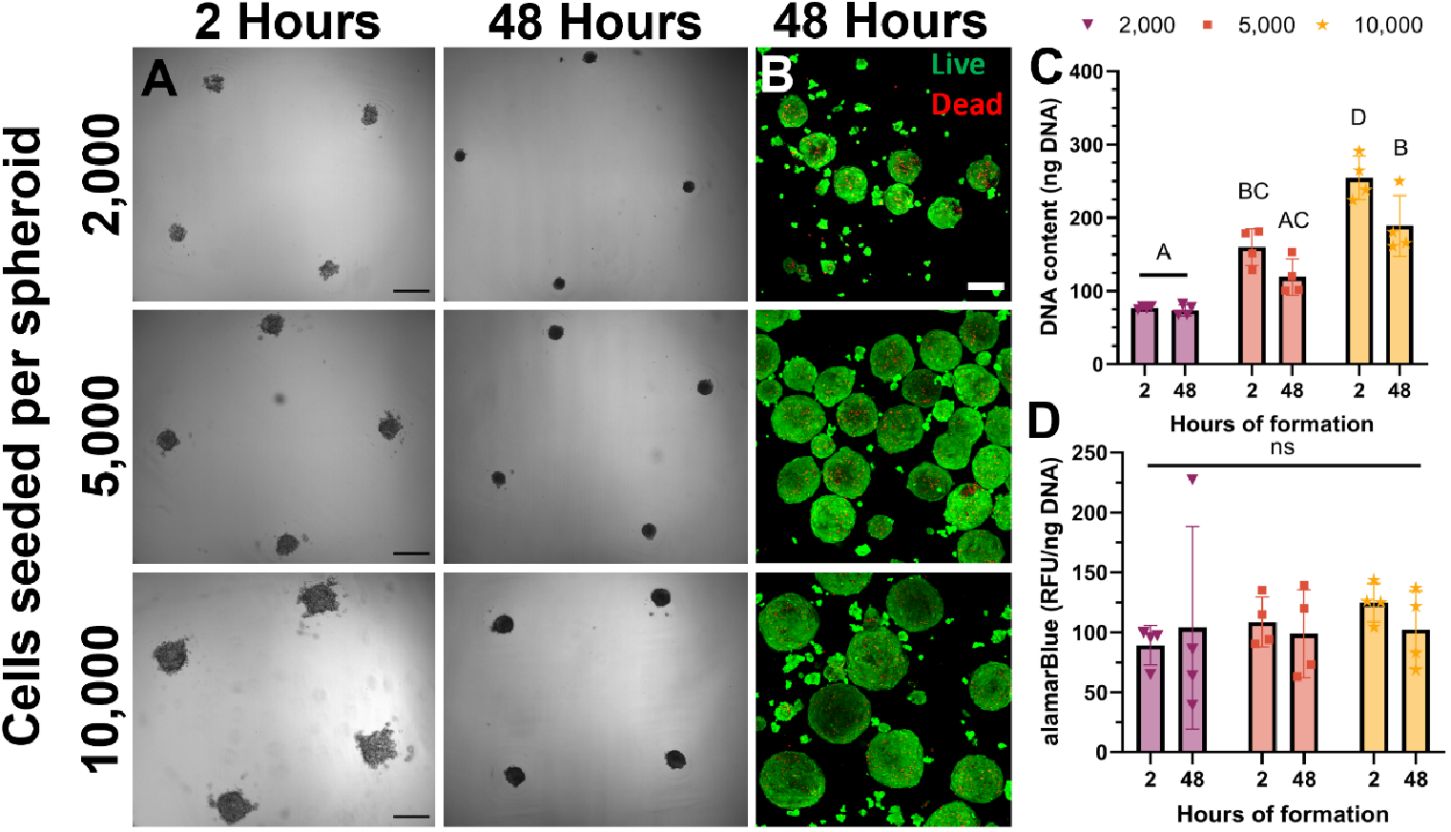
Bovine MuSCs form spheroids similar to C2C12s. (A) Bright field images of primary bovine MuSC spheroids over 48 h (scale bars represent 500 μm). (B) Live/dead confocal images of spheroids with increasing numbers of cells seeded after 48 h of formation (scale bar represents 150 μm). (C) DNA content and (D) alamarBlue quantification of bovine spheroids (*n* = 4). Groups with different letters denote significance (*p*< 0.05), while groups that share a common letter are not statistically significant.

After establishing the feasibility with C2C12s, we formed spheroids using primary bovine MuSCs to translate the findings from the murine cell line to more relevant primary cells. We formed spheroids seeded with 2000, 5000, and 10 000 cells using the same methods described for C2C12s. As with C2C12 spheroids, there was substantial compaction (Figures 5A and S3A) and no difference in viability within the first 48 h for all samples (Figures 5B and S3C). After 48 h, spheroids possessed average diameters of 146.4 ± 15.7, 193.7 ± 17.2, and 240.4 ± 19.2 μm for 2000, 5000, and 10 000 seeded cells per spheroid, respectively. Given the similarity to C2C12 spheroid formation (Figure S3B), we selected spheroids seeded with 5000 cells for bioprinting. We observed a decrease in DNA content for spheroids seeded with 10 000 cells over 48 h (Figure 5C) but no change in metabolic activity (Figure 5D). As speculated for C2C12s, cells may be attaching to the well plate underneath the agarose molds. Alternatively, the reduction in DNA content observed in spheroids seeded with 10 000 cells may result from discrepancies between cell proliferation and cell death. Cells may be dying before compacting into the spheroids, as primary cells are more sensitive than cell lines.

### 3.5 Primary Bovine MuSC Spheroids Spread and Exhibit Tissue Forming Potential

**Figure 6:**
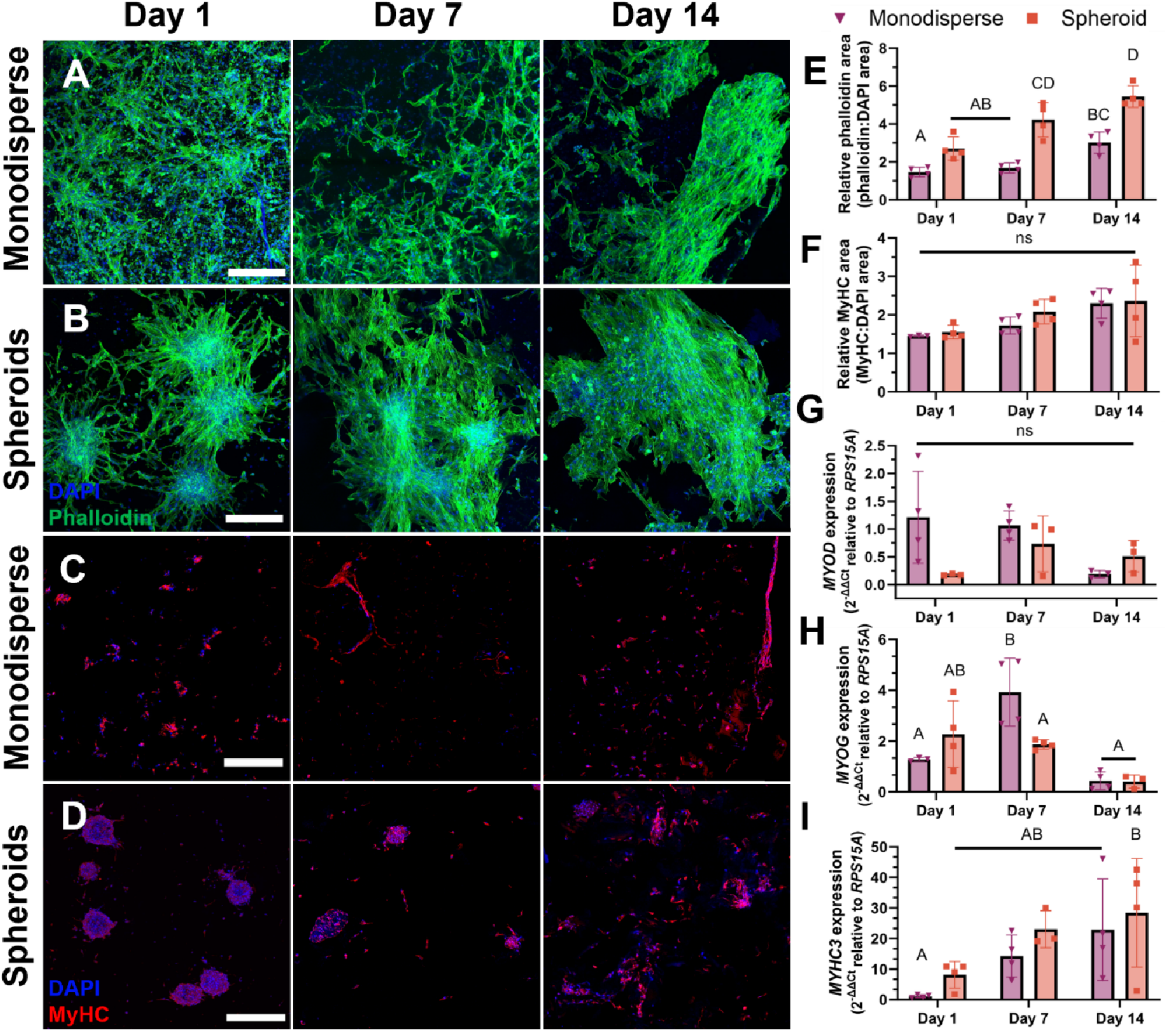
Bovine spheroids exhibit enhanced spreading and show tissue-forming potential. Confocal z-stack projection micrographs of (A) monodisperse cells and (B) spheroids bioprinted in alginate bioink and differentiated over 14 days. DAPI (blue) stain for nucleus and Phalloidin (green) for F-actin (scale bars represent 250 μm). Immunostaining of bioprinted (C) monodispersed cells and (D) spheroids with DAPI (blue) and myosin heavy chain (red) over 14 days (scale bars represent 250 μm). Quantification of the ratio of (E) phalloidin:DAPI and (F) MyHC:DAPI stain area over 14 days (*n* = 4). Quantification of (G) *MYOD*, (H) *MYOG*, and (I) *MYHC3* expression (*n* = 3–4). Data points for (E–I) represent independent samples. Groups with different letters denote significance (*p*< 0.05), while groups that share a common letter are not statistically significant.

Having determined that primary bovine MuSCs form spheroids analogously to the C2C12 cell line, we printed spheroids to compare their spreading and differentiation to monodisperse muscle stem cells. Similar to C2C12s, we observed substantial spreading with both monodisperse and spheroids after 1 day (Figure 6A,B). Monodisperse cells had an even stronger tendency to migrate toward the periphery of the construct, while the spheroids showed good spreading throughout day 14, even within the center of the sample (Figure S5). Cells at the periphery of the spheroids migrated into the surrounding matrix and began to fuse with neighboring spheroids. By day 7, we observed that cells formed bridges between spheroids, and by day 14, nearby spheroids fused into larger microtissues with minimal visibility of the initial spheroids in some regions (Figures 1B and S6). Overall, primary cells demonstrated enhanced spreading in the spheroid constructs at days 7 and 14, as indicated by Phalloidin stain area and, therefore, projected cytoskeleton area (Figure 6E). MuSCs expressed MyHC even at day 1 within both monodisperse and spheroid constructs (Figure 6C,D). Unlike the steadily increasing differentiation observed in C2C12 spheroid constructs, MyHC expression in primary muscle cell spheroids remained relatively consistent over 14 days, with no difference in protein expression between monodisperse and spheroid samples (Figure 6F). Monodisperse cell samples had decreased *MYOD* expression over 14 days (Figure 6G) along with a peak in *MYOG* expression after 7 days (Figure 6H), while spheroid samples showed no distinct temporal trends in either marker. Although not significant, *MYHC3* expression, encoding embryonic myosin heavy chain, increased steadily in both monodisperse and spheroid samples over 14 days (Figure 6I).

## 4 Discussion

Spheroids are a promising strategy for tissue engineering based on their ability to better mimic *in vivo* conditions such as cell–cell and cell–matrix interactions compared to monodisperse cells.^4,7^ Previous reports have established that both primary and immortalized muscle cells can form spheroids that promote cell viability and retain myogenic potential when cultured after dissociation.^15,16^ However, there is limited investigation into the use of skeletal muscle spheroids embedded within 3D microenvironments.^17^ Entrapment of spheroids in biomaterials enables the presentation of instructive cues that increase survival and direct cell fate while enabling implantation of cell-laden constructs for clinical translation.^10,41^ Herein, we characterized spheroids composed of C2C12 myoblasts and bovine primary MuSCs and assessed their viability, metabolic activity, morphology, and myogenic differentiation when printed in a commonly used alginate bioink.

We selected C2C12 murine myoblasts and primary bovine MuSCs due to their importance in a broad array of clinical and industrial applications. Primary cells, though more physiologically relevant, are notoriously more sensitive than established cell lines.^42^ Thus, comparing the spheroid formation and function after bioprinting in these two models may provide important translational insights. Cell lines are commonly used in the biopharma industry, and cell immortalization is a key aim of current research efforts within the field of cell-cultured meat. On the other hand, primary cells are the preferred model for regenerative medicine, where the ultimate aim is often to use autologous cell populations for various therapies. Cell-cultured meat companies are also isolating primary cells from a variety of animals to enable their work on species-specific products while they develop immortalization techniques for the reliable production of new cell lines. The verification of primary cell viability in combination with common biofabrication techniques, such as bioprinting, is critical for understanding their potential application in both cell-based clinical therapies and advances in the production of meat. Spheroids composed of both cell types remained viable after bioprinting and exhibited markers of late-stage myogenic differentiation after 2 weeks. Trends in myogenic gene expression varied between cell types, indicating that marker selection is key in following the myogenic differentiation of muscle cell spheroids in alginate hydrogels.

Spheroid diameter regulates nutrient availability to the aggregate core due to gradients within the spheroid and can have important implications on spheroid function.^10,43^ We did not observe differences in the spheroid metabolic activity with diameters below 250 μm. However, previous studies demonstrated that smaller mesenchymal stromal cell (MSC) spheroids exhibit higher levels of proliferation and metabolic activity due to greater nutrient availability, while slightly larger spheroids can secrete higher levels of trophic factors.^43^ Viability declines in cells further than 150–200 μm from oxygen and nutrient sources.^44^ The lack of differences in this study may be attributed to spheroid radii remaining under 150 μm. However, biomaterial properties and cellular metabolism may influence the effective diffusion limitations and are areas of ongoing research. Spheroids fabricated with primary bovine MuSCs formed analogously to C2C12s in terms of compaction and resulting diameter after 48 h. Given that muscle fibers of many species, including humans, are on the order of 100 μm diameter, spheroid diameter may have substantial implications on cell fusion to develop functional muscle fibers. Diameter also influences the surface area to volume ratio of cells directly interacting with the surrounding matrix. As spheroids increase in diameter, their surface area to volume ratio decreases, possibly affecting how external biophysical and chemical cues are transmitted to cells on the interior of the spheroid. We tested spheroids seeded with increasing numbers of cells from 500 to 10 000, ultimately selecting 5000 cells per spheroid to maintain a desirable surface area to volume ratio and for ease of handling.

Interspheroid spacing is an important factor dictating spheroid crosstalk and fusion.^17,45^ Unlike monodisperse cells, which are homogeneously dispersed within a material, the spacing of spheroids in biomaterial constructs is often heterogeneous.^39^ In this study, we did not measure or regulate spheroid spacing; however, others have shown that myoblast spheroids spaced approximately 300–400 μm apart achieved improved cell alignment and greater differentiation compared with smaller or larger distances.^17^ Others reported limited spreading when spheroids were more than 250 μm apart, suggesting there may be differences in spheroid fusion based on cell type, spheroid diameter, secretory profiles, and matrix interactions.^45^ Additionally, anisotropic organoid building blocks were used to demonstrate that alignment of cardiac aggregates can be enhanced by controlling morphology and shear-induced alignment during bioprinting.^24^ Taken together, these studies demonstrate that cell spheroids have substantial potential for fusion and cellular alignment. Further interrogation of factors such as spheroid spacing and diameter, total cell density, cell–cell, cell–matrix, and secretory profiles is needed to understand the interplay of these parameters.

These data confirm that muscle spheroids remain intact, viable, and rapidly spread into the surrounding matrix after bioprinting. After only 24 h, cells on the spheroid periphery exhibited substantial outgrowth toward neighboring spheroids, which fused into larger cellular structures by day 7. In some areas of the printed constructs, the spheroids fully fused into continuous microtissues after 14 days, while in other areas, spheroids remained more distinct. C2C12s exhibited no significant changes in the relative F-actin area between monodisperse and spheroid samples, yet bovine primary MuSC spheroids demonstrated more spreading compared to monodisperse MuSCs and C2C12s of either type. Monodisperse cells achieved ample spreading in early time points, but after 7 days, began to migrate toward the edges of the gels. On the other hand, the nanoporous hydrogel traps spheroid bodies in their original location. Cells on the periphery of spheroids located toward the center of the constructs displayed enhanced spreading compared with monodisperse cells in similar locations. This could be a result of upregulated cytokine production and cell–cell signaling commonly observed in spheroids, which contribute to enhanced cell survival both *in vitro* and *in vivo* compared with monodisperse cells.^4,7^ These data suggest that spheroids have the potential to fuse into microtissues at least as effectively as monodisperse cells since using spheroids in conjunction with biomaterial platforms offers a greater range of parameters that can be manipulated to induce a desired response, such as spheroid size, spacing, maturation, and growth factor loading. Though we did not see dramatic advantages with using spheroids in our study, it is possible that commonly observed benefits of spheroid culture (i.e., enhanced cell viability, local retention of growth factors, and endogenous ECM) may not have been reflected over the 14 day culture period. Due to the endogenous ECM providing a more protective environment, further engineering of muscle spheroid function could prove particularly beneficial for culturing more sensitive primary cells embedded within a 3D matrix, in a bioreactor, or during clinical use.

Interrogation of myogenic differentiation via MyHC protein expression and qPCR of genes encoding for myogenin, MyoD, and embryonic myosin heavy chain demonstrates that both cell types and culture conditions Myosin heavy chain staining revealed that the C2C12 spheroids differentiated over time in the bioprinted constructs. Alternatively, bovine muscle cell spheroids showed no significant change in MyHC signal over time but exhibited greater MyHC at each time point compared with C2C12s. As myogenic differentiation occurs, MyoD and MyoG expressions peak sequentially. Gene expression in C2C12 samples showed a reduction in Myod and no change in Myog over time, suggesting that C2C12s are likely in the early stages of myotube fusion and differentiation. Gene expression of *MYHC3*, encoding for an embryonic isoform of myosin, exhibited a more substantial increasing trend over time in bovine MuSC samples. Coupled with decreasing *MYOD* and *MYOG* that peak halfway through the experiment, this suggests that bovine MuSC samples are progressively differentiating and slightly further along than C2C12 samples.

Bovine MuSC spheroids showed a consistent trend toward increased spreading and fusion over 14 days. Monodisperse bovine MuSC samples showed minimal differences in MyHC expression over 14 days, though C2C12 cells exhibited enhanced expression on days 7 and 14 compared with day 1. Neither monodisperse nor spheroid samples of either cell type formed many distinguishable multinucleated myotubes, potentially due to the material stiffness decreasing over time. We used oxidized alginate that enables hydrolytic degradation and facilitates cell invasion into the biomaterial.^34^ Increased stiffness enhances cell spreading and myogenic differentiation,^46^ thus, matrix stiffness is likely a critical lever in tuning the fusion and differentiation of spheroids within 3D microenvironments. We previously demonstrated that the alginate bioink used in this study possessed adequate rheological properties for 3D bioprinting and enhanced print fidelity, cell–matrix interactions, and cell viability compared to alternative bioink compositions.^37^ Muscle cell differentiation is enhanced when cultured on materials with a Young’s modulus of ∼12 kPa, but the bioink used here has an initial storage modulus of ∼5 kPa. Myoblasts cultured on soft substrates exhibit self-renewal rather than differentiation.^47^ However, other biophysical properties such as hydrogel viscoelasticity, demonstrated using alginate hydrogels, can also guide myogenic phenotype.^48^ Further work is needed to develop bioinks that enable printability and cell migration while better mimicking *in vivo* stiffness and viscoelasticity. Differences between mechanotransduction pathways within spheroids and monodisperse cells are not well understood and may have significant implications for biomaterial mechanical properties and effective strategies to induce muscle cell alignment. Distinguishing the influences of spheroid diameter, morphology, degree of maturation, and differences in mechanotransduction on the differentiation of myogenic spheroids will enhance our ability to create mature, aligned muscle tissue from the myogenic spheroid building blocks.

## 5 Conclusion

This study demonstrates that C2C12 and primary bovine skeletal muscle spheroids exhibit similar spheroid formation and establishes that muscle spheroids retain viability and function after bioprinting. Overall, these data indicate that spheroids can generate 3D microtissues as effectively as monodisperse cells and, perhaps, show additional promise given that many levers can be altered to further enhance spheroid function. Compared to monodisperse cells, C2C12 spheroids show enhanced MyHC expression, while MuSC spheroids exhibit increased cell spreading, demonstrating the importance of considering spheroid culture in 3D microenvironments. Further research is warranted to better understand skeletal spheroid spreading and investigate variables that influence cell alignment, myotube formation, and myogenic differentiation.

## Supporting Information Available

Schematic of spheroid-laden bioink mixing and 3D construct formation; additional characterization of C2C12 viability; additional characterization of bovine MuSC spheroid size and viability; comparison of phalloidin and MyHC protein expression among all samples; full construct scan of DAPI/Phalloidin in bovine MuSC bioprinted samples; high magnification DAPI/Phalloidin images of bovine MuSC samples (PDF)
