# Supplementary material for: Skeletal Muscle Spheroids as Building Blocks for Engineered Muscle Tissue
Source: ACS Biomater Sci Eng. 2023 Dec 19;10(1):497–506. doi: 10.1021/acsbiomaterials.3c01078 (PMC10777344; doi:10.1021/acsbiomaterials.3c01078)
Supplement: Supplementary file 1 — ab3c01078_si_001.pdf [file ab3c01078_si_001.pdf]

Supporting information for

Skeletal muscle spheroids as building blocks for engineered muscle tissue

Nicholas Johnson[1,2], Andrea C. Filler[1,2], Akash Sethi[3], Lucas R. Smith[4], J. Kent Leach[1,2]*

[1]Department of Orthopaedic Surgery, UC Davis Health, Sacramento, CA, 95817, USA
[2]Department of Biomedical Engineering, UC Davis, Davis, CA, 95616, USA
[3]Department of Molecular and Cellular Biology, UC Davis, Davis, CA, 95616, USA
[4]Department of Neurobiology, Physiology and Behavior, UC Davis, Davis, CA, 95616, USA

Corresponding Author:

J. Kent Leach, Ph.D.
Department of Orthopaedic Surgery
UC Davis Health
4860 Y St., Suite 3800
Sacramento, CA, 95817
+1-916-734-8965
jkleach@ucdavis.edu

Keywords: spheroids, skeletal muscle, bioprinting, muscle engineering, hydrogel

Supplementary figures

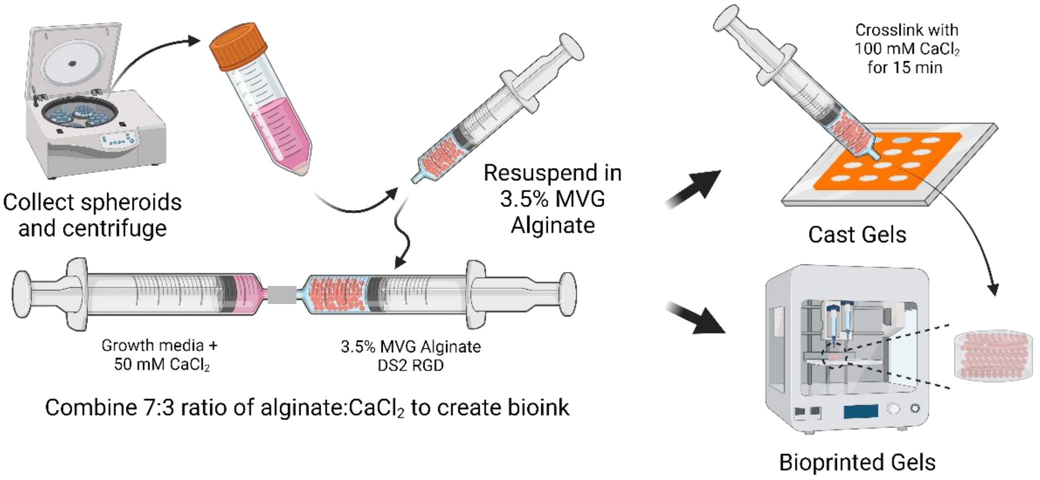

Figure S1. Schematic of process to form spheroid-laden alginate bioink and cast or bioprinted gels.

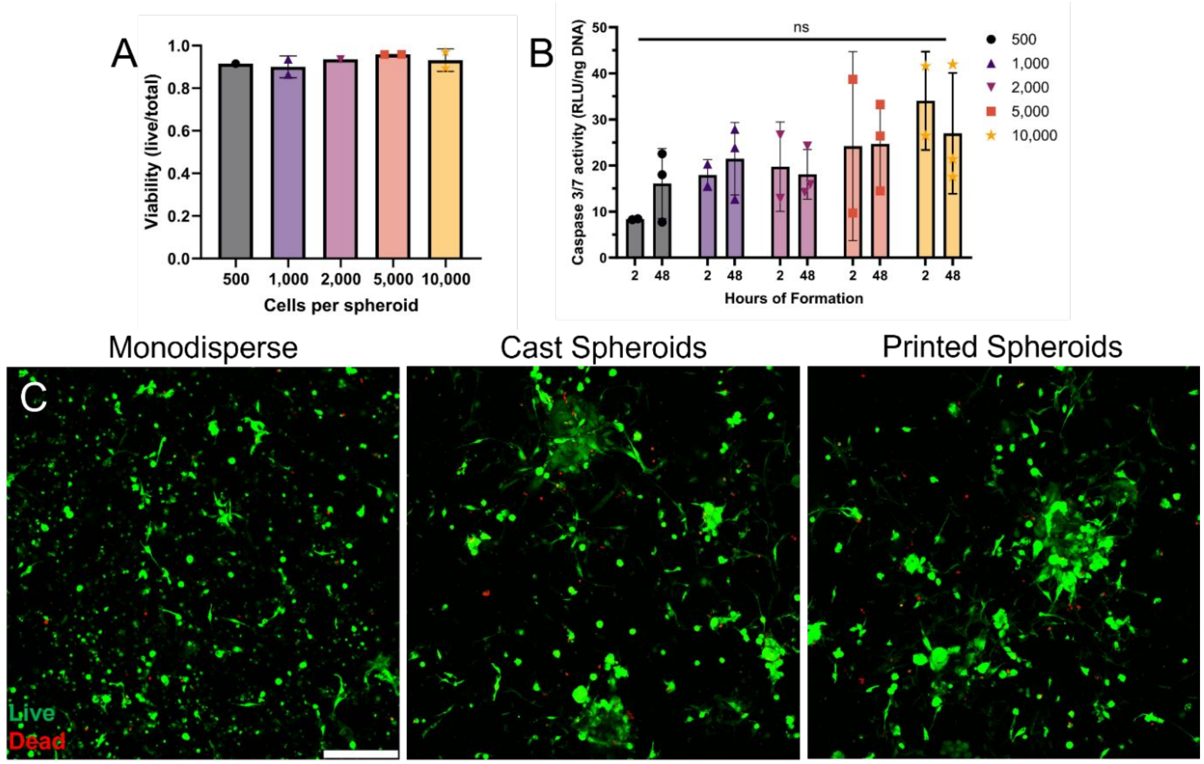

Figure S2. Additional characterization of C2C12 viability. (A) Quantification of live/dead for spheroids of increasing cell density (n=1,2,1,1,2). **(B)** Caspase 3/7 quantification as a marker of apoptotic activity (n=3). **(C)** Quantification of live/dead assay after spheroid bioprinting at days 3 & 7. **(D)** Confocal images of live/dead assay 7 days after bioprinting (scale bar represents 200 μm). Groups with different letters denote significance (p<0.05), while groups that share a common letter are not statistically significant.

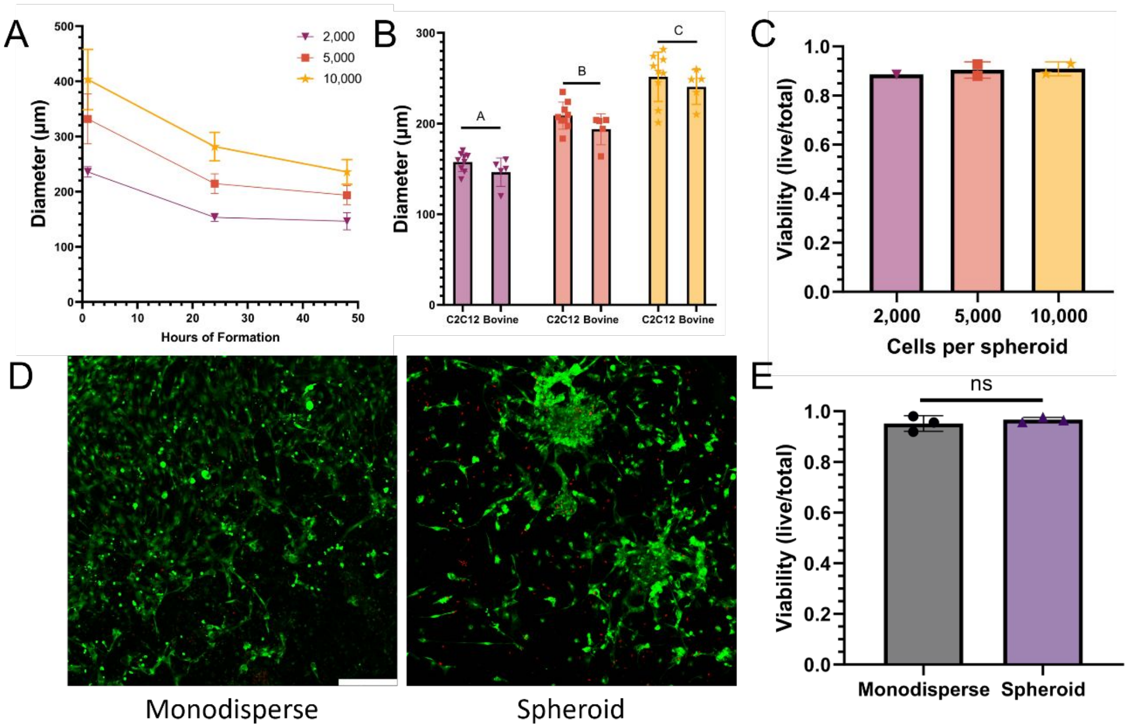

Figure S3. Additional characterization of bovine spheroids and viability of bovine MuSCs after bioprinting. (A) Bovine spheroid compaction over 48 hours and (B) comparison of C2C12 and bovine spheroid diameters after 48 hours. **(C)** Quantification of bovine spheroid live/dead images for increasing cell densities (n = 1,2,2). **(D)** Confocal live/dead images of monodisperse bovine MuSCs and spheroids after bioprinting (scale bar represents 200 µm) and **(E)** quantification of those images (n=3). Groups with different letters denote significance (p<0.05), while groups that share a common letter are not statistically significant.

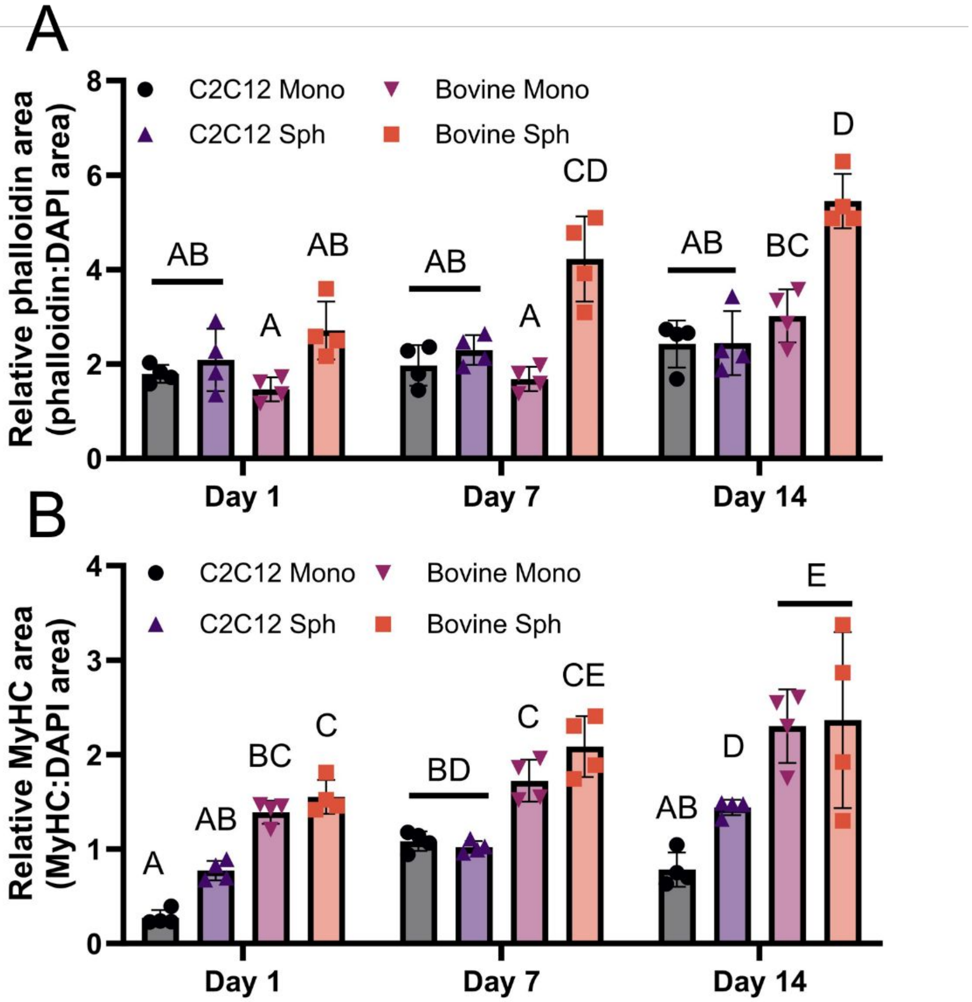

Figure S4. Spheroid culture enhances function of bovine MuSCs in bioprinted alginate constructs. Comparison of all quantification for **(A)** phalloidin:DAPI and **(B)** MyHC:DAPI ratios in both cell types over 14 days (n=3-4). Groups with different letters denote significance (p<0.05), while groups that share a common letter are not statistically significant.

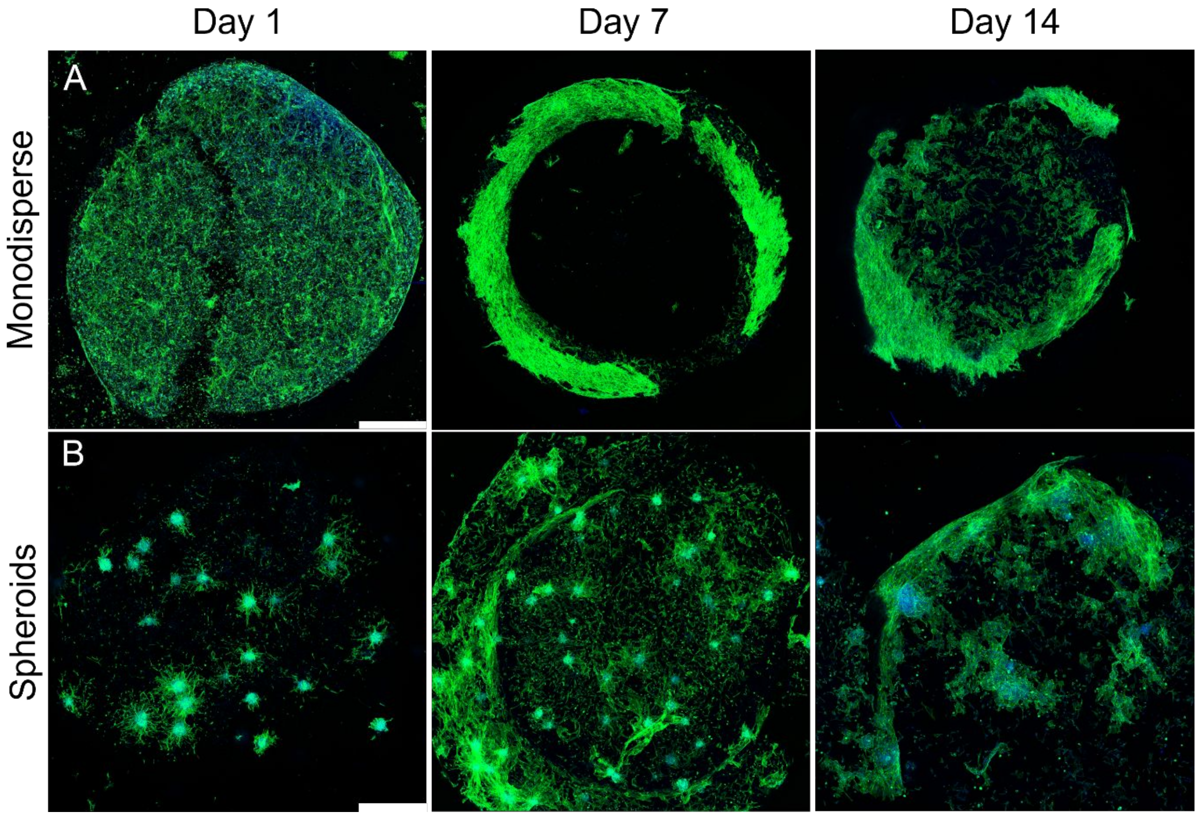

Figure S5. Full construct scan of primary muscle cells printed in oxidized alginate. Confocal z-stack projection micrographs of full bioprinted constructs differentiated over 14 days containing **(A)** monodisperse cells and **(B)** spheroids in alginate (scale bars = 750 µm). DAPI (blue) stain for nucleus and Phalloidin (green) for F-actin.

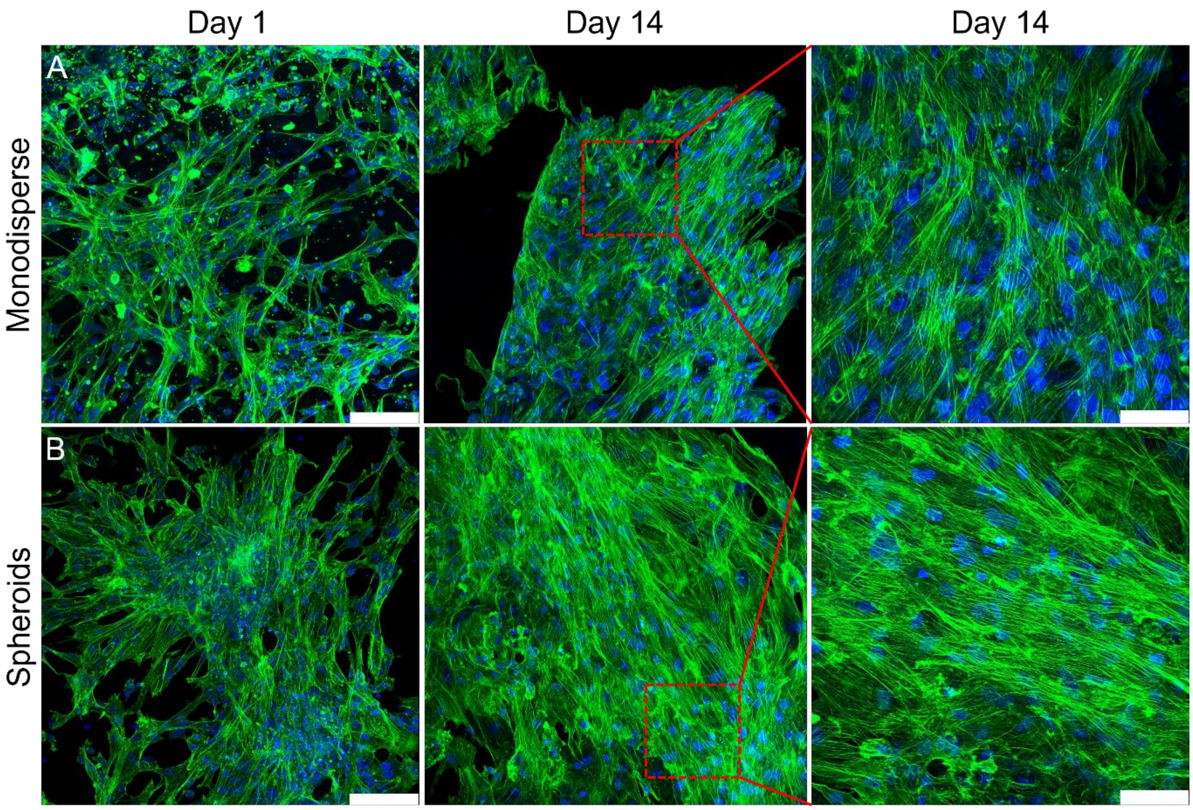

Figure S6. High magnification view of bioprinted bovine samples. Confocal z-stack max projections of DAPI/Phalloidin-stained **(A)** monodisperse and **(B)** spheroids bioprinted in alginate. (Scale bars = 100 µm and 50 µm for Day 14 insets)
